# A class frequency mixture model that adjusts for site-specific amino acid frequencies and improves inference of protein phylogeny

**DOI:** 10.1186/1471-2148-8-331

**Published:** 2008-12-16

**Authors:** Huai-Chun Wang, Karen Li, Edward Susko, Andrew J Roger

**Affiliations:** 1 Department of Biochemistry and Molecular Biology, Dalhousie University, Halifax, N.S. B3H 1X5, Canada; 2 Department of Mathematics and Statistics, Dalhousie University, Halifax, N.S. B3H 3J5, Canada; 3 Centre for Comparative Genomics and Evolutionary Bioinformatics (CGEB), Dalhousie University, Canada

## Abstract

**Background:**

Widely used substitution models for proteins, such as the Jones-Taylor-Thornton (JTT) or Whelan and Goldman (WAG) models, are based on empirical amino acid interchange matrices estimated from databases of protein alignments that incorporate the average amino acid frequencies of the data set under examination (e.g JTT + F). Variation in the evolutionary process between sites is typically modelled by a rates-across-sites distribution such as the gamma (Γ) distribution. However, sites in proteins also vary in the kinds of amino acid interchanges that are favoured, a feature that is ignored by standard empirical substitution matrices. Here we examine the degree to which the pattern of evolution at sites differs from that expected based on empirical amino acid substitution models and evaluate the impact of these deviations on phylogenetic estimation.

**Results:**

We analyzed 21 large protein alignments with two statistical tests designed to detect deviation of site-specific amino acid distributions from data simulated under the standard empirical substitution model: JTT+ F + Γ. We found that the number of states at a given site is, on average, smaller and the frequencies of these states are less uniform than expected based on a JTT + F + Γ substitution model. With a four-taxon example, we show that phylogenetic estimation under the JTT + F + Γ model is seriously biased by a long-branch attraction artefact if the data are simulated under a model utilizing the observed site-specific amino acid frequencies from an alignment. Principal components analyses indicate the existence of at least four major site-specific frequency classes in these 21 protein alignments. Using a mixture model with these four separate classes of site-specific state frequencies plus a fifth class of global frequencies (the JTT + cF + Γ model), significant improvements in model fit for real data sets can be achieved. This simple mixture model also reduces the long-branch attraction problem, as shown by simulations and analyses of a real phylogenomic data set.

**Conclusion:**

Protein families display site-specific evolutionary dynamics that are ignored by standard protein phylogenetic models. Accurate estimation of protein phylogenies requires models that accommodate the heterogeneity in the evolutionary process across sites. To this end, we have implemented a class frequency mixture model (cF) in a freely available program called QmmRAxML for phylogenetic estimation.

## Background

Since the 1970s, the evolution of protein sequences has been modelled using empirical amino acid substitution matrices derived from analyses of databases of protein alignments. Since the first introduction of these 'accepted point mutation' (PAM) models by Dayhoff and coworkers [1], a variety of newer substitution matrices have been derived based on much larger databases of alignments (e.g. the JTT matrix [2], the BLOSUM family [3], the probability matrix from blocks (PMB) [4], WAG [5]) or databases of proteins encoded by specific genome types (e.g. mitochondria [6] and chloroplasts [7]) and using more rigorous statistical methods (see [8,9] for a recent discussion).

However, it has long been recognized that different sequence positions evolve at different rates. Indeed, a significant improvement in the fit of these models to real data has been to model heterogeneity in rates at different sites using a discrete approximation to the gamma distribution [10]. Yet it is well known that sites in proteins not only differ in their relative rates of evolution, but, because of structural and functional constraints, they also differ in their preferences for specific amino acids. Some sites in a protein alignment are occupied by almost any residue, while others appear to be restricted to a limited subset of amino acids and, quite frequently, only one particular residue. Attempts at improving substitution matrices for database searching, take these forms of substitution heterogeneity into account with the development of position-specific scoring matrices [3], profile-based methods [11], hidden Markov models [12] and structure-specific substitution matrices [13]. However, accounting for site- or structure-specific dynamics in amino acid replacements in protein phylogenetic models has only recently garnered significant attention.

Bruno [14,15] proposed a model where site-specific amino acid frequencies were estimated by maximum likelihood (ML). However, this model is problematic because the number of parameters increases without bound (19 per site) [16] and a large number of taxa are required for model fitting. Goldman and coworkers [17,18] introduced a set of eight to ten predefined categories of substitution patterns at sites in a hidden Markov model framework, based on protein secondary structures and surface exposure, and each category has its own rate matrix for ML inference. Other models explore the interdependence of sites due to constraints introduced by tertiary structure in protein sequence evolution [19-21]. Lartillot and Philippe [22] proposed a Bayesian mixture model that allows amino acid replacement pattern at different sites to be described by distinct substitution processes which have the same substitution rates but different stationary probabilities. They implemented their CAT model in a Bayesian Monte Carlo framework with a Dirichlet process prior. More recently Le et al. [23] proposed a new amino acid profile mixture model in which substitutions at sites follow a 'proportional' model whereby site-specific substitutions are entirely characterized by a mixture of 10 to 60 equilibrium frequency classes at sites.

Lartillot, Le and colleagues [22-24] argue that taking into account the site-specific nature of protein evolution may be of vital importance to phylogenetic estimation especially in the case where two or more branches are extremely long, leading to an apparent long branch attraction (LBA) type artefact in empirical data sets. They, and a recent study by Rokas and Carroll [25], have shown that 'homoplasy' (i.e. multiple independent origins of the same character state at a homologous site in different taxa) occurs much more frequently in true protein alignments than expected under standard substitution models such as JTT or WAG, even when the rates-across-sites process is taken into account. As a result, they suggest that even probabilistic methods (i.e. maximum likelihood or Bayesian methods) employing these standard models can display an LBA bias, even when a large number of sites are considered. Lartillot's CAT model and the methods proposed by Le *et al*. are designed to counter these problems, although at the cost of a large number of additional parameters to be estimated and some model simplifications.

Here we revisit the issue of site-specific amino acid constraints in protein phylogenetics. First, to further probe the differences between the 'true' substitution process and standard models, we assembled 21 large protein sequence alignments and used two different methods to test if and how empirical frequencies at sites differ from those simulated under the standard JTT + F + Γ substitution model. We showed that significant deviations can be detected for the majority of these protein families. Second, using the site-specific amino acid frequencies estimated from one of the data sets, we simulated a four-taxon case over a large grid of different branch-length settings to evaluate the accuracy of the ML methods employing a standard empirical matrix to recover the correct tree under these conditions. We found a large 'Felsenstein zone' where the LBA artefact occurs. Third, we conducted a principal components analysis of the amino acid frequency matrix at all sites of the 21 protein alignment data sets and obtained four major classes (or profiles) of amino acid frequency distribution at sites. We propose a random effects mixture model using these class frequencies to model site-specific amino acid frequency distributions and implemented it in a version of RAxML [26] that we call Q-matrix mixture RAxML (QmmRAxML) for phylogenetic inference. This model differs from previous models [22,23] by accounting for intrinsic exchangeabilities between amino acids and containing standard amino acid substitution models as a special case, thereby permitting likelihood ratio testing of improvements in model fit. We show that the amino acid frequency mixture model fits the data significantly better than the conventional non-mixture model in all cases examined and further find it can reduce the LBA artefact both in simulations and in a phylogenomic analysis of a eukaryotic data set. In comparison to other approaches, our model introduces significantly fewer additional parameters and avoids model over-simplification.

## Results and discussion

### Statistical analyses of site-specific amino acid uniformity and state frequencies

An entropy-based measure was used to quantify the deviation of site-specific amino acid frequencies from uniform usage of amino acids. A Z-test was used to determine whether the real data are more or less uniform compared to a very large data set simulated under JTT + F + Γ for the same tree. A total of 21 protein data sets (numbers of taxa and sites shown in Table 1) and associated simulated data (100,000 amino acid sites for every simulated data set) were used and the sites of the real and simulated data were divided into four estimated rate categories and Z-tests were carried out on each rate category. The P-values for the tests are shown in Table 1. These indicate that for the sites in the slowest rate category (rate 1) less than half of the datasets have significant differences in amino acid uniformity between the real data and the simulated data; but for sites in the faster rate categories (rates 2–4), the real data are less uniform than the simulated data in the majority of the cases.

**Table 1:** Statistical analyses of site-specific amino acid uniformity and state frequencies in 21 protein data sets.

Protein family	Taxa	Sites	Z-test (uniformity)				χ^2 ^test (states)
				
			Rate 1	Rate 2	Rate 3	Rate 4	
Carboxyl_trans	36	212	0.97	**	0.05	*	**
CTP-synthetase	65	212	**	**	**	**	*
DNA topo IV	49	228	0.21	**	**	**	*
Filament	36	210	0.35	0.09	0.92	0.45	0.66
Glu_synth_NTN	40	253	**	**	**	**	0.01
HSP70	34	432	0.31	**	*	**	**
ILVD_EDD	51	310	0.20	*	**	**	**
MCM	40	220	0.66	*	*	0.11	**
MreB	32	275	0.50	0.10	**	*	0.03
Poty_coat	34	212	0.19	**	**	**	**
SecA	70	203	**	**	**	**	**
Usher	36	317	*	**	**	**	0.08
HSP90	54	459	**	**	**	**	**
NuoF	41	405	**	**	**	**	**
Cpn60	41	466	0.18	0.04	**	**	**
MPP	43	203	0.04	0.24	**	0.03	0.32
α-tubulin	54	375	**	*	**	**	*
β-tubulin	46	382	**	**	*	**	0.02
Actin	48	363	**	**	**	*	*
EF-1α	38	361	0.29	**	**	**	**
EF-2	37	669	**	**	**	**	**

P-values: ** < 0.001; * < 0.01. The protein family abbreviations are: Carboxyl_trans, acetyl-CoA carboxylase; Cpn60, 60-kDa chaperonin; DNA topo IV, DNA topoisomerase IV subunit A (GyrA); EF-1α, elongation factor 1α; EF2, elongation factor 2; Filament, intermediate filament protein; Glu_synth_NTN, Glutamate synthase aminotransferase; HSP70, 70-kDa heat shock protein; HSP90, 90-kDa heat shock protein; ILVD_EDD, dehydratase family proteins; NuoF, NADH dehydrogenase I chain F; MCM, minichromosome maintenance protein; MPP, mitochondrial processing peptidase sequences; MreB, a bacterial homolog of the eukaryotic actin; Poty_coat, potyvirus coat protein; Usher, Fimbrial usher protein.

**Figure 1:**
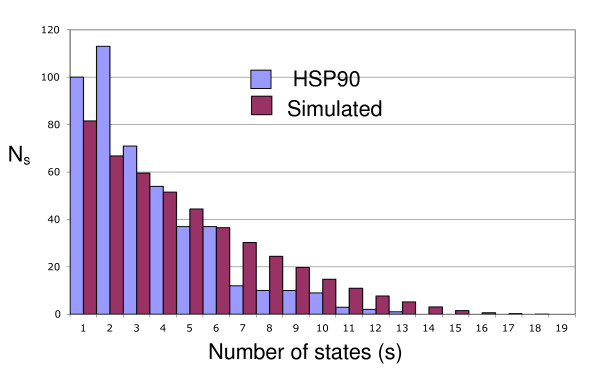
**Numbers of sites with a given number of states in simulated versus real HSP90 data**. The original HSP90 data have 54 taxa and 459 sites. The simulated data have the same number of taxa and 100,000 sites. In the latter case the proportions of sites with each number of states were calculated and then multiplied by 459 to make the numbers directly comparable to the HSP90 data set.

Table 1 also shows the results of the state frequency tests. For these analyses a χ^2 ^test was used to compare the numbers of sites with a given number of observed states in real data (observed counts) versus those in simulated data (expected counts). Only three data sets showed no significant differences in the amino acid state frequency counts between the real and simulated data. For the remaining 18 data sets, the real data and the simulated data have very (P < 0.001) or moderately (P < 0.05) significant differences in the number of distinct amino acids at the sites. Moreover, in all these cases, the simulated data have greater numbers of distinct amino acid states at sites. Figure 1 shows the distribution of the number of sites with a given number of states for the heat shock protein 90 (HSP90) data set compared with the data simulated using the HSP90 tree under the JTT + F + Γ model with parameters fit to the data set. Relative to the simulated data, the real HSP90 data set has more sites with fewer states and no sites that have more than 13 states. By contrast, the simulated data set has sites with as many as 18 observed states.

Collectively, these tests indicate that real data sets tend to have less uniform amino acid frequencies and fewer states at sites than expected under standard phylogenetic substitution models such as JTT + F + Γ.

### Four-taxon tree simulations under a site-specific frequency model and average frequency of the whole data set

**Figure 2:**
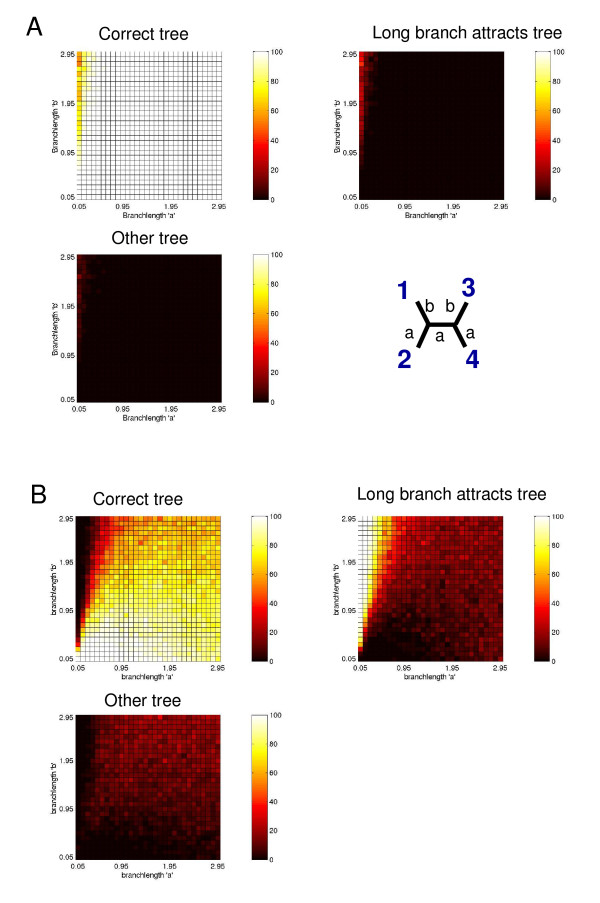
**Performance of ML tree reconstructions evaluated using simulations**. The performance of ML tree reconstruction with the JTT + F + Γ model for data simulated under (A) the JTT + F + Γ model and (B) under a site-specific frequency model (JTT + ssF + Γ). The site-specific frequency data were derived from the HSP90 data set. The three heatmaps in (A) and (B) represent, respectively, the proportions of "Correct tree" (i.e., taxa 1 and 2 together), "Long branch attracts tree" (i.e., taxa 1 and 3 together) and "Other tree" (i.e., incorrectly put taxa 1 and 4 together) with regard to branch-lengths *a *and *b*. The four-taxon tree shown in (A) is the true tree (taxa 1 and 2 together, and taxa 3 and 4 together) used for simulating the data. Each box of the heatmaps represents 100 simulations for the given conditions.

In order to evaluate the potential impact of restricted site-specific amino acid frequencies on ML-based phylogenetic inference we did simulations of four-taxon trees under an extreme 'site-specific frequency' (ssF) model. For this model, the amino acid frequencies at each site were calculated from the HSP90 data set. These frequencies were then used in a JTT + ssF + Γ model to simulate data sets over the four-taxon trees over a wide range of branch-length settings. Note that for this model, each site is simulated with stationary frequencies corresponding to the frequencies observed in a given HSP90 alignment column. For each branch-length setting, trees were then estimated by ML under JTT + F + Γ with Tree-Puzzle [27] (Fig. 2B). For comparison, we also simulated data sets for the same branch-length settings using the average amino acid frequencies observed in the whole HSP90 data set using the standard JTT + F + Γ model. Trees for these latter simulated data sets were also estimated by ML under the JTT + F + Γ model to evaluate a case where there is no model misspecification (Fig. 2A). The results show that for the data simulated under the JTT + ssF + Γ model but with phylogenies estimated under the JTT + F + Γ model there is a very serious LBA bias such that the two taxa with long branches (taxa 1 and 3) group together (Fig. 2B upper right graph). Moreover, the fraction of other incorrectly estimated trees (i.e., taxa 1 and 4 group together; Fig. 2B lower graph) is also quite high. In contrast, the LBA topology and the third incorrect topology are much less frequently estimated from the data simulated under the JTT + F + Γ model (Fig. 2A), although a small LBA bias is observed.

### Principal Components Analysis of site frequency data

**Figure 3:**
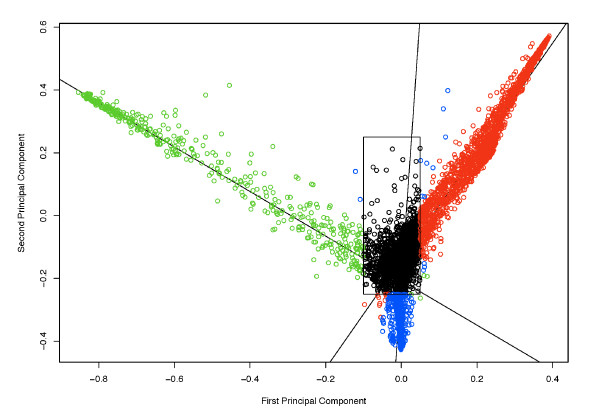
**Principal components analysis of the amino acid frequency matrix from 21 protein data sets**. Each site is indicated by an open circle. The classes and the regression lines were determined as shown in the main text.

**Figure 4:**
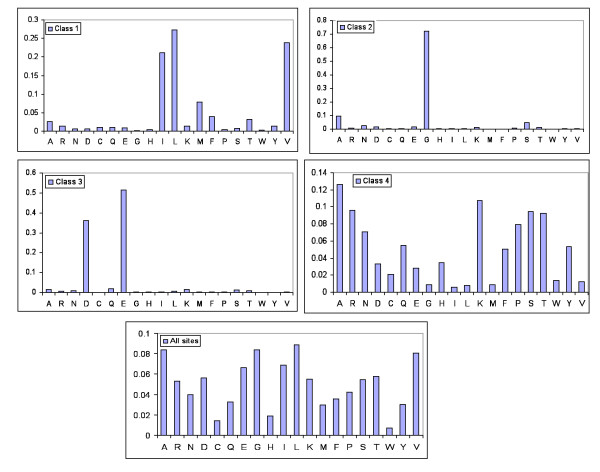
**Average amino acid frequencies in the four site-specific classes derived from the PCA shown in Figure **3. The bottom frequency profile shows the overall frequencies of amino acids observed at all sites in the 21 amino acid alignments.

The above simulation results show definitively that, if ignored, site-specific amino acid frequencies will cause significant LBA problems for phylogenetic inference. Although Bruno (1996) [14] attempted to account for this phenomenon by modelling amino acid frequencies at each site of the alignment, this approach leads to a serious statistical problem [16] whereby the number of model parameters increases linearly with the amount of data. If, however, certain patterns of amino acid usage are recurrent, one may derive some common frequency vectors, or profiles, from a large number of sequence sites. To determine whether there were such recurrent patterns in the data, we calculated the 20 amino acid frequencies from each of the 6555 sites of the 21 protein data sets to form a matrix of 6555 sites × 20 frequencies. We then carried out a principal components analysis (PCA) and plotted the first two components (Fig. 3). These two components account for 21.6% of the variance of the data. We used a clustering method to cut the distribution of the sites into four classes along the lines of linear regressions (see Methods for details). The first class has high frequencies of valine, isoleucine and leucine, followed by methionine. The second class has high frequencies of glycine, followed by alanine and serine. The third class is rich in aspartic acid and glutamic acid. The fourth class, a 'left-over' class that contains those sites that are not obvious members of the first three classes, has much more uniform frequencies of the various amino acids. The predominant amino acids in the first three classes are consistent with the observation that the amino acids in those classes are biochemically and structurally similar and expected to be more interchangeable over evolutionary time. Figure 4 shows the average amino acid frequencies of the four PCA-derived classes as well as the overall average amino acid frequencies of the 21 data sets.

### Testing a class frequency mixture model

Using the four amino acid frequency profiles from the PCA analysis and adding a fifth one corresponding to the average frequency of the whole data set to account for site frequencies not captured by the PCA classes and also to make the relevant model comparisons easier (see the Methods section for details), we implemented a 'class frequency' (cF) mixture model. In this model, the site likelihood is calculated as a weighted sum of the site likelihoods conditional on each class frequency or the whole data set frequency separately. We also account for rates-across-sites using standard discretized gamma mixture model methods. The cF mixture model has been implemented in QmmRAxML, based on the source code of the phylogenetic inference package RAxML.

We used QmmRAxML to calculate likelihoods of 25 protein data sets under the single frequency model (JTT + F + Γ) and the cF mixture model (JTT + cF + Γ), respectively. The first 21 data sets are the same ones that were used for deriving the class frequencies from the PCA, while the last four data sets are additional protein alignments used to test the generalizability of the cF model to other data sets. For the analyses under the cF mixture model, we fixed the tree topologies to be the same optimal trees recovered under the JTT + F + Γ model but branch-lengths were re-optimized. QmmRAxML uses an Expectation-Maximization (EM) algorithm to optimize weights of the class frequencies and the whole data set frequency. The estimated weights (the w_c _parameters in the model described in the Methods section) and the likelihood differences (ΛlnL) between the two models are listed in Table 2. In all cases there are significant likelihood increases under the cF mixture model compared to the single frequency model, indicating the cF model always fits the data better for the same topology. Curiously, in all cases the weight of the F class (the average frequency of the whole data set) is generally high and the weights of the four PCA classes are generally low, especially in classes 2 and 3. The weight for a class estimates the probability that a site has a frequency vector corresponding to that class. The reasons for the relatively small class 1–3 weights are several-fold. First, it seems likely that these frequency classes are related to structural features. For instance, the two major amino acids of class 3, aspartic acid and glutamic acid, are negatively charged and many sites that are conserved to have only these amino acids could have important structural roles such as binding metal cations, participating in intramolecular and/or intermolecular salt-bridges, or have catalytic or substrate-binding functions. Although the number of such sites will vary across protein families, it is likely that they will always constitute a relatively small minority in any given protein family. The data bears this out in other ways. Most sites that do not have a very pronounced preference for the amino acids emphasized in classes 1–3 or that have appreciable frequencies for more than four amino acids, will be fit best by either class 4 or the F class. Since classes 1–3 emphasize sites with very few amino acids each of which occur with frequencies of < 10% overall, it is not surprising that the estimated frequency of these kinds of sites are all quite low. For example, aspartic acid and glutamic acid together have an average frequency of 12.2% in the 21 data sets. Therefore, the sites restricted to having virtually only these two amino acids, as featured in class 3, are expected to be very small and is reflected by the low weights assigned to this class.

**Table 2:** Fitting the class frequency mixture model (JTT + cF + Γ) to 25 protein data sets.

Protein	Taxa	Sites	w(Π_F_)	w(Π_1_)	w(Π_2_)	w(Π_3_)	w(Π_4_)	ΛlnL
Carboxyl_trans	36	212	0.74	0.11	0.06	0.00	0.10	67.16
CTP-synthetase	65	212	0.28	0.29	0.13	0.04	0.24	225.24
DNA topo IV	49	228	0.58	0.15	0.05	0.02	0.21	162.77
Filament	36	210	0.81	0.10	0.00	0.05	0.05	39.58
Glu_synth_NTN	40	253	0.66	0.13	0.04	0.01	0.17	76.31
HSP70	34	432	0.65	0.17	0.02	0.0002	0.16	136.71
ILVD_EDD	51	310	0.65	0.14	0.06	0.01	0.14	181.56
MCM	40	220	0.65	0.18	0.03	0.00	0.14	74.38
MreB	32	275	0.52	0.20	0.07	0.00	0.22	141.87
Poty_coat	34	212	0.60	0.17	0.04	0.02	0.18	125.57
SecA	70	203	0.40	0.24	0.09	0.08	0.19	217.82
Usher	36	317	0.78	0.10	0.02	0.004	0.10	76.11
HSP90	54	459	0.37	0.19	0.05	0.09	0.30	279.92
NuoF	41	405	0.37	0.20	0.11	0.04	0.27	186.40
Cpn60	41	466	0.52	0.19	0.04	0.03	0.22	257.04
MPP	43	203	0.73	0.13	0.03	0.00	0.11	74.82
α-tubulin	54	375	0.46	0.16	0.04	0.01	0.33	90.05
β-tubulin	46	382	0.59	0.15	0.03	0.02	0.21	69.84
Actin	48	363	0.58	0.12	0.03	0.02	0.25	41.50
EF-1α	38	361	0.60	0.15	0.05	0.00	0.21	104.78
EF-2	37	669	0.52	0.16	0.06	0.03	0.22	273.30

enolase	60	305	0.63	0.13	0.06	0.00	0.19	24.08
myoglobin	80	153	0.59	0.14	0.06	0.03	0.17	35.73
lipoprotein	23	762	0.77	0.10	0.02	0.01	0.10	70.70
lysozyme	36	127	0.61	0.12	0.03	0.02	0.23	18.23

ΛlnL is the likelihood difference between the cF mixture model and the single frequency model (JTT + F + Γ). The p-values associated with these differences, calculated from χ^2 ^tests with 4 degrees of freedom, are very significant in all cases (p < 0.01). The actual p-values would be even smaller as the tests are conservative (see the main text for a discussion).

**Figure 5:**
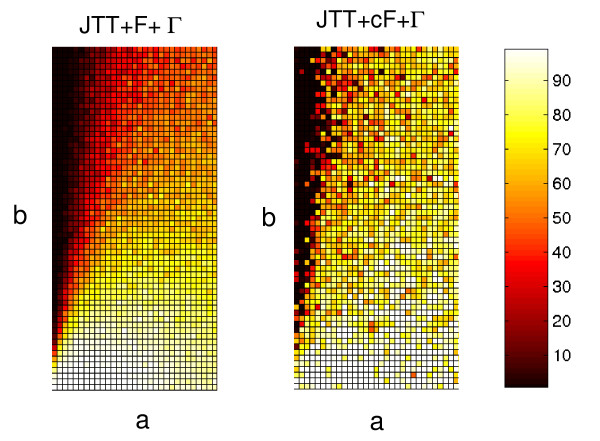
**The performance of ML tree reconstruction with the JTT + F + Γ model and the JTT + cF + Γ model**. The data were simulated under the site-specific frequency model (JTT + ssF + Γ) based on amino acid frequencies observed at each site of the HSP90 alignment. The ranges of branch-lengths *a *and *b *are 0.05–1.45 and 0.5–2.95, respectively, with an increment of 0.05. The left and right heatmaps represent, respectively, the proportions of correctly estimated trees estimated under JTT + F + Γ and JTT + cF + Γ models. Each box of the heatmaps represents 100 simulations for the given conditions.

In the above simulation studies we have demonstrated that sequence data generated under the JTT + ssF + Γ model can cause a serious LBA problem when the trees are estimated under the conventional JTT + F + Γ model. To test whether the cF mixture model can ameliorate the LBA problem we used both simulations and analysed a real data set. Figure 5 shows the simulation results from data generated under JTT + ssF + Γ model. The left panel shows the results of estimation under a standard JTT + F + Γ model and the right panel shows estimation under the JTT + cF + Γ model. From comparing these two results, it is clear that the cF model ameliorates some, but not all of the LBA problems relative to the standard model. It is interesting to note that in the non-Felsenstein zone region, the cF model sometimes performs slightly worse than the standard model. This is consistent with higher variance estimates expected from a model that includes more parameters.

The deep phylogeny of eukaryote 'supergroups' is often plagued with LBA [28], even when large multi-gene phylogenomic data sets are used [29]. One of the most famous examples of this concerns the position of Microsporidia, a group of fast-evolving intracellular parasites that are now known to be relatives of Fungi [30]. When reconstructing the phylogeny of eukaryotes rooted by Archaea with conventional models, the extremely long branch leading to Microsporidia is often attracted to the long branches leading to the Archaea at the base of the eukaryotes. Many methods have been proposed to solve this problem, including selective taxon sampling, removal of fast-evolving proteins and saturated sites [28,29], accounting for covarion shifts [28], amino acid profile mixture modelling [23], *etc*. Here we applied the cF mixture model to a large set of eukaryote phylogenomic data with 133 proteins from 40 taxa and 24294 sites [29] and calculated likelihoods of two competing trees: the LBA topology where Microsporidia group with Archaea and, the correct topology where Microsporidia group with Fungi. As shown in Table 3, while the JTT + F + Γ model supports the Microsporidia plus Archaea clan, the cF mixture model supports the correct Microsporidia-Fungi tree. Thus for a real example the cF model appears to be less susceptible to the effects of LBA than the standard model.

**Table 3:** Analysis of a large phylogenomic data set [29] consisting of 133 proteins from 40 taxa, 24294 sites for two competing trees under single frequency model and cF mixture model.

Tree	Single frequency model (JTT + F + Γ)	Class-frequency mixture model (JTT + cF + Γ)
*Microsporidia plus archaea clan*	-745,292.15*	-738,445.15
*Microsporidia plus Fungi clade*	-745,366.62	-738,371.59*

### Comparisons to other methods

Lartillot and colleagues have shown that accounting for site-specific amino acid frequencies with their CAT + Γ model seems to significantly improve both model fit and phylogenetic estimation with large concatenated protein data sets [24]. Indeed, they were the first to observe that accounting for site-specific frequencies is important to avoid long-branch attraction problems when there are large numbers of substitutions (i.e. so-called 'substitutional saturation'). However, as these authors later note, the standard CAT + Γ model fits very large numbers of classes that, while appropriate for large concatenated data sets, likely leads to problems with over-parameterization and convergence of Bayesian analyses in the case of smaller alignments (e.g., less than 1000 sites) [23].

To address this, Le and colleagues [23] developed similar models based on a set of 10 to 60 classes of amino acid profiles that were estimated from a database of alignments and implemented these in both Bayesian (PhyloBayes) and maximum likelihood (PhyML) estimation programs. However, again, for computational efficiency reasons, the authors restricted attention to proportional models that ignore different intrinsic 'exchangeabilities' between amino acids. This, and the fact that weights associated with the 10 to 60 classes should be estimated for every tree, suggests that problems may still exist with both over-simplification of the substitution process (i.e. ignoring exchangeabilities), overparameterization of the models and computational efficiency.

Our model is developed with more of a 'bottom-up' style approach; we have introduced very few frequency classes, that, based on our PCA of a set of real alignments, seem to be the most important amino acid profiles. Furthermore, we use the exchangeabilities from standard models (e.g. JTT or WAG) and include the overall frequencies as one of the classes. This setup allows nested model comparisons for likelihood ratio tests to directly examine improvement in model fit by the introduction of new classes.

It is interesting to compare this approach to that described by Le and colleagues [23]. Le and colleagues have estimated a larger number of frequency classes directly by the method of maximum likelihood from a large database of alignments and therefore these classes in principle should fit the data they were estimated from better than the PCA approach described here. However, because of the assumption of uniform exchangeabilities, they are likely to estimate too many classes some of which are distorted frequency classes that adjust for increased exchangeabilities present in more complex and realistic rate matrices than the uniform matrix. Our approach may avoid this situation and therefore may require fewer frequency classes to adequately capture the site-specific nature of protein evolution. Furthermore, by including the data set frequencies as a fifth component, sites that show little preference for a restricted amino acid profile, but evolve according to the exchangeabilities of the JTT matrix can be accommodated.

Nevertheless, despite the improvements in phylogenetic estimation we have found, the four classes we introduced from the PCA may in fact be too few to adequately capture the diversity of 'site-specific' preferences in amino acids. For instance, we did not recover a class where the basic amino acids lysine and arginine predominate, even though sites that rapidly switch between these amino acids are clearly observable in protein families [31]. The fact that the first two components of our PCA account for only ~21% of the variance in the data indicates that inclusion of additional classes by investigation of the third or more principal components from the PCA or other data mining methods such as the self-organizing map [32] may be fruitful future directions to take. In any case, our implementation of these methods in a generic Q-matrix mixture model in QmmRAxML, allows the user to implement any number of exchangeability matrices plus associated stationary frequency vectors to freely explore improvements in model fit in protein evolution.

## Conclusion

We report the results of two statistical tests – the amino acid frequency uniformity test and state counts test – that demonstrate that in real protein alignments there are fewer states at sites and the frequencies of these states are less uniform than predicted by JTT + F + Γ model. We show that use of standard 'average' frequency models like JTT + F + Γ for phylogenetic estimation when the data are simulated with site-specific frequencies leads to serious LBA artefacts. A PCA of site-specific frequency vectors of 6555 sites from 21 protein data sets revealed four major classes of sites. These classes can be used in a simple class-frequency (cF) mixture model for modelling site-specific distributions for phylogenetic inference that we have implemented in a program called QmmRAxML. Likelihood ratio tests indicate a large improvement in the fit of JTT + cF + Γ over JTT + F + Γ for all data sets examined. Furthermore, the cF mixture model appears to ameliorate the long-branch attraction problems, in both simulation studies and in analyses of a phylogenomic data set. The cF mixture model provides a new method for modelling site-specific compositional heterogeneity and QmmRAxML is a promising tool for exploring model fit in protein evolution and reconstructing more accurate phylogenies.

## Methods

### Data sets

To obtain a representative set of protein alignments with enough taxa and sites to test for departures from the empirical JTT amino acid substitution model, we took the 7459 seed alignments from Pfam-A database (release 14.0) and filtered it using two criteria. First, only alignments with > 30 sequences were considered and submitted to the Gblocks program [33] to automatically trim regions of ambiguous alignment with a minimum block size set to 5 and maximum number of contiguous nonconserved positions of 16. From this set of trimmed alignments only data sets with > 200 positions were considered, yielding a final set of only 12 alignments. To these 12 alignments, 9 alignments of proteins used for phylogenetic studies in our laboratory were added that met the requirement of > 30 taxa and > 200 sites after trimming with Gblocks (using the same settings as above). The 21 protein families examined are indicated in Table 1 and include proteins with functional roles ranging from components of the cytoskeleton (e.g. tubulins and actin), to globular enzymes (e.g. CTP-synthetase) to viral coat proteins (e.g. Poty_coat).

For each of the 21 data sets, a phylogenetic tree was estimated under the JTT + F + Γ model with 8 gamma rate categories using Tree-Puzzle (version 5.2). The resulting trees were used to simulate amino acid sequences of 100, 000 sites under JTT + F + Γ using Seq-Gen [34].

### Statistical analyses

Method 1 – Amino acid uniformity and uniformity test: We utilized an information theoretical notation of relative entropy (*r*), also called the Kullback-Leibler divergence [35], to measure the amino acid uniformity at sites. It is defined as:

$$\text{r}=\log20+\sum_{\text{i}=1}^{20}{\text{P}}_{\text{i}}\cdot \log{\text{P}}_{\text{i}}$$

where *P*_*i *_is the frequency of amino acid *i *at a given site. A site with all 20 AA's having equal frequencies (P_i _= 0.05) would have an *r *= 0; a perfectly conserved site would result in the maximum possible *r *= log20 = 4.32 bits; all other sites would have an *r *between 0 and 4.32 bits.

The uniformity test asks whether the amino acid frequencies at sites in real data have the same uniformity as those in data simulated under the JTT + F + Γ model. An $\bar{\text{r}}$ averaged over all sites is calculated respectively for a real data set (${\bar{\text{r}}}_{\text{real}}$) and for a corresponding data set simulated under JTT + F + Γ (${\bar{\text{r}}}_{\text{JTT}+\text{F}+\Gamma }$). The simulated data have 100,000 sites, so the standard error of ${\bar{\text{r}}}_{\text{JTT}+\text{F}+\Gamma }$ is effectively 0 and therefore can be ignored allowing a simple z-test. The test statistic for the uniformity test is a *z*-score defined as

$$z_{r}=\frac{{\bar{r}}_{real}-{\bar{r}}_{JTT+F+\Gamma }}{s_{real}/\sqrt{n}}$$

where S_real _is the standard deviation of *r*_*real *_and *n *is number of sites in the real data.

Furthermore, the sites of the real and simulated data were divided into four rate categories and Z-tests were conducted on each rate category separately.

Method 2 – State frequency test: comparing the number of states at a site in real data and in simulated data. The test statistics is defined as

$${\text{c}}^{2}=\sum_{\text{y}=1}^{20}\frac{{\left({\text{o}}_{\text{y}}-{\text{e}}_{\text{y}}\right)}^{2}}{{\text{e}}_{\text{y}}}$$

where O_y _is the number of sites showing *y *distinct character states observed in real data and e_y _is expected number of sites showing *y *distinct character states in simulated data under JTT + F + Γ. The c^2 ^has an approximate χ^2 ^distribution with 19 degrees of freedom.

### Simulations of four-taxon trees under a site-specific frequency model

We sought to evaluate the potential impact of site-specific amino acid frequencies on ML-based phylogenetic inference with empirical amino acid substitution models and overall data set frequencies. To do this, site-specific amino acid frequencies were calculated from the HSP90 protein family and these frequencies were used to simulate data sets of four sequences using covTREE (morticia.cs.dal.ca/lab_public/?Download:covTREE), a C++ adaptation of Seq-Gen. The simulation settings were as follows.

Following the studies of Huelsenbeck [36] and Wang et al. [37], we evaluated tree reconstruction efficiency, over a grid of branch-lengths *a *and *b*, for trees of the form of ((1:a,2:b),(3:a,4:b):b). In the figures, the grids of branch-lengths have *a *on the x-axis and *b *on the y-axis with an increment of 0.1 for both *a *and *b*, within a range of 0.05 to 2.95 substitutions per site. For each branch-length setting implied by a given element of the grid, 100 simulated data sets were generated. For evaluation of the class frequency model described below, similar grids were calculated but with finer increments of 0.05, and focussing on the 'Felsenstein zone' region. In this case the range of *a *was 0.05–1.45 and the range of *b *was 0.5–2.95.

### Principal components analysis of amino acid frequency at sites

An amino acid frequency composition vector was calculated for every site in each of the 21 protein data sets and assembled into a matrix of 6555 sites by 20 amino acid frequencies. To investigate whether there were any recurring patterns in these frequency vectors, principal component analysis was performed using the R package [38]. The four site classes, given in Figure 3, were determined as follows. An initial clustering divided sites into three classes based of the first principal component (less than -0.04, greater than 0.04 on the x-axis, or between these bounds). For each cluster, linear regression applied to the first two principal components gave the three lines in Figure 3. Sites were then classified to whichever line they were least distant from. To reduce the risk of misclassification, sites with first principal component between -0.1 and 0.05 and second principal component between -0.25 and 0.25 were excluded from class frequency calculation associated with the linearly determined classes; this gave a fourth class. The aggregate amino acid frequencies of these four classes (i.e., the site frequency profiles) were calculated and used in the class frequency model (see below).

### A class frequency (cF) mixture model

We proposed a class frequency mixture model under which the likelihood of a sequence site is a weighted sum of the site likelihood conditional on each of the class frequencies found from the PCA analysis. In order to take account of frequency distributions not modelled in the PCA study of the limited data (the 21 data sets), the cF mixture model was further added with a fifth class that corresponds to the average amino acid frequency of the whole data set. The cF model can further be combined with a Γ model to take account of the rates-across-sites variation and the site likelihood under a JTT + cF + Γ is given in the following equation.

$$\text{L}({\text{x}}_{\text{i}})=\frac{1}{g}\sum_{\text{c}=1}^{5}{\text{w}}_{\text{c}}\sum_{\text{k}=1}^{\text{g}}\text{P}({\text{x}}_{\text{i}}|{\text{r}}_{\text{k}},\Pi_{\text{c}})$$

where x_*i *_are data at site *i*, the w_c_'s are the probabilities (i.e., weights) of the class frequencies, including the whole data set frequency as one class, r_k _is the rate of a Gamma distribution discretized into one of the *g *categories with equal probabilities.

In this likelihood calculation the usual JTT + F + Γ model is a special case of the JTT + cF + Γ mixture model when the probability of each of the class frequency profiles is 0 and the probability of the whole data set frequency profile is 1. Therefore, a likelihood ratio test (LRT) may be used to compare the models, where the test statistics is twice the difference in log-likelihoods of the alignment under the two models and a P-value can be calculated from a χ^2 ^distribution with 4 degrees of freedom. However, since the parameters (i.e., the weights of the cFs) are on the boundary of the parameter space, a simple χ^2 ^approximation does not hold. The real distribution of the test statistics follows a mixture of χ^2 ^distributions [39,40] and the P-value is even smaller. If this were the only complication, then the P-values reported using the χ^2 ^distributions would be conservative estimates.

However, an additional complication in calculating degrees of freedom arises because the proteins for which a comparison between the JTT + F + Γ and JTT + cF + Γ models was desired were sometimes also used in constructing the class frequencies, although this is not true for the four additional alignments at the bottom of Table 2. An extremely conservative estimate in these cases would adjust the degrees of freedom upwards by 19 × 4 = 76. In this case, a difference in log likelihoods for the two models would be declared significant at the 5% level if it exceeds 51. Table 2 indicates that this is the case for all but two of the 21 proteins used in constructing the class frequencies. This adjustment, however, greatly understates the significance of the differences. For a given alignment, an additional 76 degrees of freedom would be appropriate if the class frequencies were chosen to give the largest likelihood for that alignment. Not only are the class frequencies not chosen to give largest likelihoods but they are based on 21 different alignments, making it unlikely that any one alignment would have a substantial influence on them.

The cF mixture model was implemented in a maximum likelihood framework for phylogenetic inference, by modifying the source code of RAxML-VI-HPC version 2.2.3 [26] to produce the 'Q matrix mixture RAxML' or QmmRAxML for short. As the weights for the class frequency profiles are not known, an Expectation-Maximization algorithm [41,42] (described in the following subsection) was used to optimize the weights from an initial set of equal weights. In addition to modelling a mixture of site class-frequency profiles as discussed in this paper, QmmRAxML may also be used to model any mixture of amino acid substitution matrices, such as those based on protein secondary structures and solvent accessibilities at sites.

### Parameter optimization and the Expectation-Maximization algorithm

In QmmRAxML we use an alternating scheme to optimize parameters in the class frequency mixture model, including branch-lengths, the among-sites rate variation parameter (α) and the weights (w_c_) of class frequency vectors. First the program has the branch-lengths and α optimized with routines in the original RAxML for an initial set of w_c_, which set all weights equal. Then it uses an EM algorithm to optimize w_c _for the current branch-lengths and α. Then it optimizes branch-lengths and α again and followed by updating w_c _with another round of an EM. These processes repeat until a maximum likelihood is reached for the current topology.

In updating the w_c_'s each round of EM itself alternates between performing an expectation (E) step, which computes a conditional expectation, conditional upon the data, of the complete likelihood by including the latent variables (w_c_) as if they were known, and a maximization (M) step, which computes the maximum likelihood estimates of the parameters by maximizing the expected likelihood found on the E step. The parameters found on the M step are then used to begin another E step, and the process is repeated. Specifically the EM updating scheme for the w_c_'s is as follows.

Let L_ci _be the likelihood for the *i*th site fixing the *c*th class frequency vector and L_i _be the overall likelihood at the current weight parameters for the *i*th site. At the *j*th iteration,

$$\begin{array}{cc} {\text{L}}_{\text{i}}=\sum_{\text{c}=1}^{\text{k}}{\text{w}}_{\text{c},\text{j}}\times {\text{L}}_{\text{ci}} & \left(**\right) \end{array}$$

where k = 5 is the number of class frequency vectors plus the average frequency vector of the whole data set.

Then the updating scheme is

$$\begin{array}{cc} {\text{w}}_{\text{c, j}+1}={\text{w}}_{\text{c},\text{j}}\times \sum_{\text{i}=1}^{\text{n}}\frac{{\text{L}}_{\text{ci}}}{{\text{L}}_{\text{i}}}\times \frac{1}{\text{n}} & \text{(*)} \end{array}$$

where n is the number of the sites. The program continues updating w_c _according to (*) and (**) until they converge.

## Availability and requirements

* Project name: A class frequency mixture model for protein phylogeny

* Project home page: http://www.mathstat.dal.ca/~hcwang/QmmRAxML/

* Operating system(s): Any Unix/Linux platform

* Programming language: ANSI C

* Other requirements: GCC (version 3 or higher) or compatible compiler

* License: GNU public license version 2

* No restrictions on use

## Authors' contributions

AJR conceived of the study, directed the analyses and drafted an outline of the manuscript. H-CW and KL contribute equally to this work. H-CW developed the QmmRAxML software, with KL carried out the analyses and with AJR drafted the manuscript. KL assembled the data sets. ES contributed statistical expertise to the analyses, conducted the principal components analyses and downstream site-classification, helped in software development and edited the manuscript.
